# Integration of CCT/TRiC assembly, degradation and function: Chaperonin complexity in a cellular context

**DOI:** 10.1016/j.cstres.2026.100207

**Published:** 2026-08-21

**Authors:** Julie Grantham

**Affiliations:** Department of Chemistry and Molecular Biology, University of Gothenburg, Göteborg, 40530, Sweden

**Keywords:** Molecular chaperone, Cytoskeleton, Cancer, Aggregation, Proteostasis

## Abstract

The essential molecular chaperone CCT/TRiC, found in the cytosol of all eukaryotes, has long been established as the molecular chaperone required for folding the abundant cytoskeletal components actin and tubulin. Advances in single-molecule imaging, cryoelectron microscopy, and the availability of extensive omics data contribute to revealing the functional scope of this multi-subunit assembly. This review focuses on how the regulation of CCT assembly and degradation of subunits contributes to the complex interplay between CCT oligomer and monomeric subunit functions and the implications of this for cancer cell biology and during impaired proteostasis.

## Background

The complex and coordinated activity of molecular chaperones contributes to the essential process of maintaining proteostasis. The intricate balance between protein synthesis, protein folding, secretion, and degradation is critical to cellular health. Molecular chaperones, isomerases, and proteases, which together form the protein quality control machinery, are tasked with ensuring the correct folding of proteins and the degradation and clearance of misfolded proteins. How on-pathway intermediate folding states are distinguished from off-pathway misfolded proteins and how chaperones aid in the folding of thousands of structurally distinct proteins present intricate challenges involving a complex interplay between components of the protein quality control machinery.

Nascent polypeptide chains and folding intermediates may receive non-specific assistance from general molecular chaperones such as the Hsp70/40 systems, where binding motifs consisting of hydrophobic amino acid residues present on the surface of non-native proteins are recognized.^1^ This provides a general mechanism to detect non-native proteins and provides a mechanism to shield sequences that may be prone to aggregation. Further folding support (often considered to be post-translational) is provided by the chaperonin subfamily of the molecular chaperones. The chaperonins share varying degrees of sequence similarity with each^2^ and are barrel-shaped assemblies formed from two rings of protein subunits. The bacterial chaperonin GroEL functions together with a co-chaperone GroES, to encapsulate folding substrates and is categorized as a Group I chaperonin together with chaperonins from chloroplasts and mitochondria. The chaperonins of archaea and eukaryotes (termed Group II chaperonins) function without a co-chaperone and instead have a helical protrusion extending from each subunit, which together can close access to the central cavity.^3^

Many *E coli* proteins are able to bind to GroEL, presumably via hydrophobic regions exposed in non-native conformations,^4^ approximately 80 proteins are catagorized as obligate substrates, suggesting some selectivity or substrate-specific dependence during folding.^5^, ^6^, ^7^, ^8^ GroEL/GroES can provide non-specific folding assistance to proteins of less than approximately 80 kDa by encapsulation.^9^ Encapsulation occurs within the chaperonin folding cavity, where the inside of the chaperonin cavity provides a binding interface capable of recognizing non-native proteins. Nucleotide-dependent conformational changes induce changes in the surface of the cavity, and this is thought to drive folding reactions.^10^ Interestingly, it has been reported that substrates bound within the GroEL cavity can be destabilized and that a reduction in the hydrophobic effect contributes to this, raising the possibility that such effects can extend to other situations where proteins are encapsulated.^11^ If similar effects occur for the folding substrates of other chaperonins remains to be determined.

The chaperonin found in all eukaryotes, chaperonin containing tailless complex polypeptide1 (CCT), also called the tailless complex polypeptide 1 ring complex (TRiC), presents a complex binding interface. Unlike GroEL, which consists of two rings of seven identical subunits, CCT consists of eight distinct protein subunits, each the product of an essential gene.^12^ The abundant cytoskeleton components actin and tubulin are dependent upon CCT for folding^13^ and are thus defined as obligate folding substrates.

## The CCT oligomer: Folding mechanisms, substrates, and impaired function

**Fig. 1:**
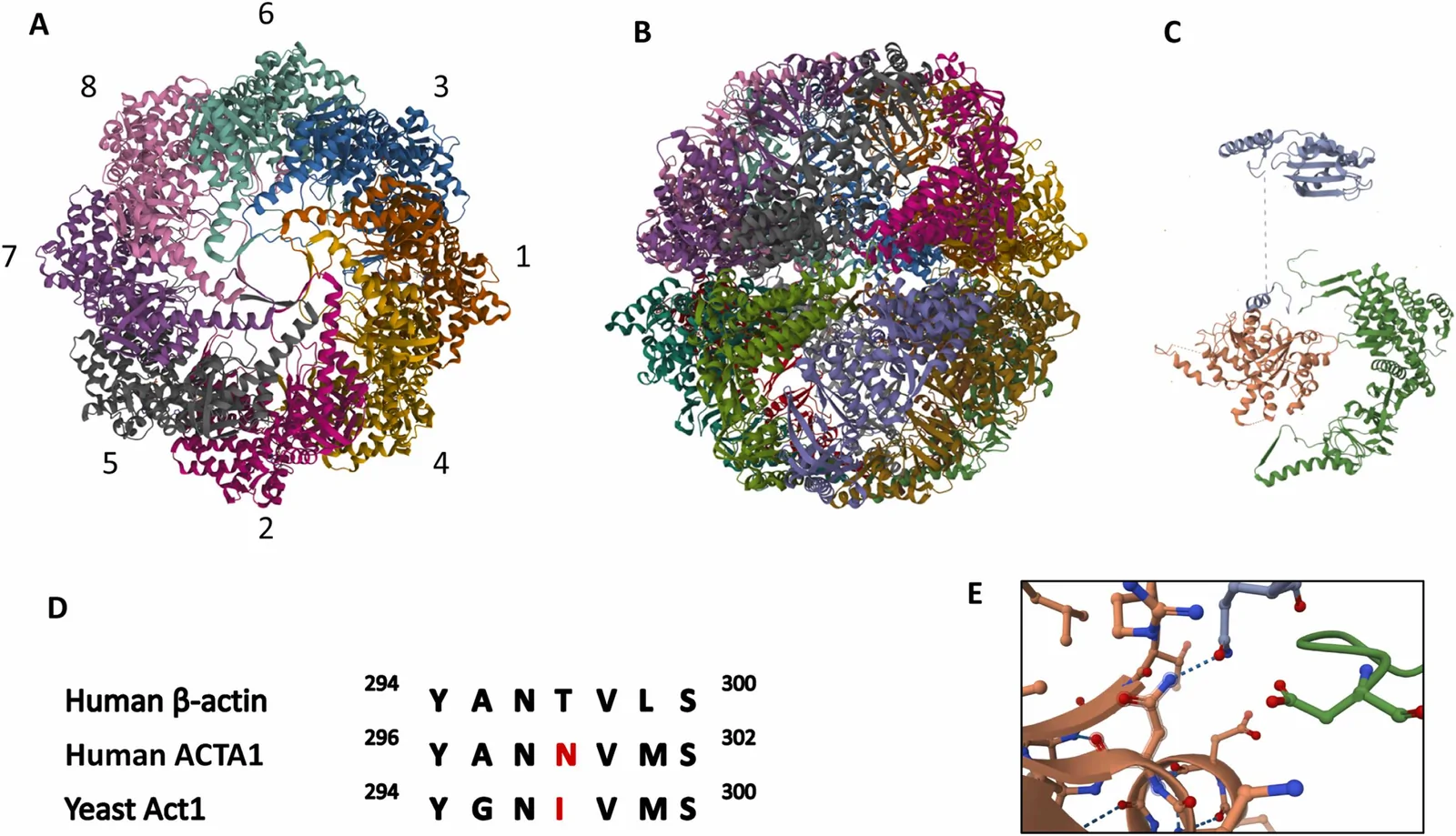
*Structural overview of the CCT oligomer and structural context of species specificity of CCT-mediated actin folding.* (A) The CCT subunit order within a single ring (PDB-7NVM). (B) Side view of the CCT oligomer (PDB-7NVM). (C) view of PhLP2A (grey), β-actin (terracotta), and CCT7 (green) (PDB-7NVM), note that the PhLP2A spans across both rings of the chaperonin. (D) Sequence alignments of human β-actin ACTB (P60709), human skeletal muscle α-actin ACTA1 (P68133) and yeast actin ACT1 (P60010) with the amino acid residues responsible for conferring species specificity shown in red. (D) zoomed in view of actin (terracotta), PhLP2A (grey) and CCT7 (green) (PDB-7NVM). Actin N296 (highlighted) forms a hydrogen bond with PhLP2A Q2, in close proximity to D528 of CCT7.

Cryo-electron microscopy has been fundamental for understanding the folding mechanisms of CCT. Initially, three-dimensional reconstructions of actin:CCT and tubulin:CCT complexes established that both actin and tubulin bound to CCT in a conformation spanning the central cavity.^14^, ^15^, ^16^ This implied that the geometry of binding was important, where specific CCT subunits occupying fixed positions within the chaperonin ring presented a substrate binding interface with specific docking sites for different folding substrates. Critical to understanding the folding mechanisms of CCT was the establishment of the CCT subunit order within each chaperonin ring^17^, ^18^ (Figure 1A) and the discovery that the CCT subunits are not equal with regard to ATP. It was observed that a mutation at a conserved site involved in ATP hydrolysis had different effects in yeast, depending on which CCT subunit contained the mutation.^19^ It was then determined that the CCT subunits different affinities for ATP, resulting in an asymmetry of ATP-binding within the folding-active chaperonin ring.^20^ Thus, it can be considered that fundamental to the folding of at least actin and tubulin, geometrically defined interactions with specific CCT subunits, together with an ATP-generated asymmetric power stroke, provide the basis for folding.

Since the first cryo-electron microscopy three-dimensional reconstruction of actin bound to CCT was obtained,^16^ numerous studies have aimed to define the folding cycles of actin and tubulin. Recently, these efforts have culminated in reconstructions in the range of 2.5–3.8 Å. Using a purification tag, the CCT oligomer bound to endogenous substrates was isolated and treated with ADP-AlFx with the aim of isolating CCT oligomers in later stages of the folding cycle.^21^ This approach not only generated CCT:tubulin complexes but also CCT:actin:PhLP2A complexes where the PhLP2A co-chaperone density extends from the non-folding chamber through to the folding chamber, where it is described as a “molecular strut”^21^ (Figure 1B and C). Interestingly, a density was observed at the septum dividing the two chaperonin chambers, and the hypothesis is presented that this region of the CCT oligomer will be involved in substrate binding.^21^

Reconstitution approaches using purified proteins to generate CCT:tubulin:prefoldin complexes have enabled the visualization of substate handover from prefoldin to CCT and define four folding intermediates of tubulin.^22^ The domains of tubulin are folded in a sequential manner: first the N-terminal domain, then the C-terminal domain, maturation of the GTP binding pocket and then the mid-domain, where electrostatic interactions between CCT and tubulin are observed with all four folding intermediates.^22^ These results are consistent with earlier models proposed where CCT-driven folding reactions use electrostatic, subunit-specific interactions to enable the folding of the obligate folding substrates [reviewed by^23^].

The interplay between prefoldin:CCT and PhLP2A:CCT is explored in reconstitution experiments using both cryo-electron microscopy and biochemical analyses, where it is suggested that prefoldin and PhLP2A may compete for binding to CCT.^24^ It was demonstrated that the prefoldin (which appeared to bind to CCT4) was readily displaced from CCT by PhLP2A (which displayed multivalent binding), whilst prefoldin had a much reduced ability to displace PhLP2A from CCT.^24^ This could thus represent a mechanism whereby competitive binding of these co-chaperones promotes a sequential folding pathway. The folding mechanisms of CCT arising from cryo-electron microscopy studies are the focus of several recent review articles.^25^, ^26^, ^27^

Interplay between CCT and prefoldin is further explored in a single-particle tracking approach designed to study interactions in cultured mammalian cells.^28^ Here, it was observed that prefoldin prolongs the interaction time of nascent actin with CCT as well as during post-translational folding interactions.^28^ The actin appeared to be able to cycle on and off CCT, whilst the CCT often remained in close proximity to the polysomes, leading the authors to conclude that CCT may maintain low-affinity interactions with either polysomes or chaperones.^28^ Such interactions would minimize the risk of partially folded actin interacting non-productively or aggregating with other proteins in the complex and crowded cellular environment. The concept of actin cycling between CCT-bound and free forms during folding but remaining in close proximity to the CCT may reflect rapid rebinding to CCT if the actin has failed to reach the native conformation. Such a mechanism may be critical for ensuring the correct forward folding of actin along what can be viewed as a ‘one way track’ as it is known that during unfolding experiments, actin must be stabilized if it is to be able to rebind to CCT and not collapse into an irretrievable misfolded conformation.^29^, ^30^

For actin and tubulin, the most abundant obligate folding substrates, there is now a great deal of structural and mechanistic information regarding the folding cycles available. However, certain questions remain, such as why does actin need CCT to reach the native state, yet the structurally similar bacterial protein MreB folds in the absence of CCT [reviewed by^31^]. The species incompatibility observed between CCT and actin (yeast CCT can fold human β-actin, but not human skeletal α-actin^32^, ^33^) offers insights into the specificities of folding, and the single substitution of N297I within human skeletal α-actin renders it foldable by yeast CCT.^32^ Interestingly, this region of actin, when viewed in the structural context of the interaction between human CCT, actin, and PhLP2A,^21^ is found to be in close proximity to the C-terminus of CCT7 and the contact between actin and PhLP2A (hydrogen bond between N296 of actin and Q2 of PhLP2A) (Figure 1C to E). It is possible that the substitution of the polar asparagine to the non-polar isoleucine has the potential to affect interactions with both CCT7 and the chaperone cofactor PhLP2A, thus accounting for the species specificity of CCT folding actin.

The extent of the folding substrate interactome of CCT has been the subject of numerous studies, and there is controversy regarding CCT: substrate specificity. In addition to actin and tubulin being obligate substrates, Cdc42 and Cdh1 also require interactions with CCT to reach their correct folded state.^34^ Studies to define the interactome of the CCT oligomer present data sets indicating that numerous proteins may bind to the CCT oligomer,^35^, ^36^, ^37^, ^38^ although such interactions may represent opportunistic binding if off-pathway folding has occurred, rather than a folding substrate that is dependent upon CCT for folding. It is also important to note that the overlap between the substrates identified in these studies is rather low. The high abundance of actin and tubulin ultimately leads to these proteins representing a large proportion of the proteins bound by the CCT oligomer^35^, ^39^, ^40^ and this is seen quantitatively in.^21^ An approach to apply a quantitative assessment to the yeast protein interactome also defined a rather discrete interactome for the CCT oligomer, supporting the view that CCT has a restricted array of folding substrates.^41^

Cryo-electron tomography was utilized to assess conformations and occupancy of the CCT oligomer in cultured human cells.^42^ Of those oligomers that had density consistent with bound substrate, only approximately 12% had prefoldin bound,^42^ consistent with prefoldin being involved in handover of substrates to CCT rather than remaining bound as a co-chaperone throughout the CCT folding cycle. Whilst the substrate was only bound in one cavity and not both, the open and closed state of the rings was not asymmetric, with a distribution of approximately 55% closed and 32% open conformations observed.^42^ Furthermore, the majority of CCT oligomers had densities consistent with bound substrate, indicating that the CCT oligomer is functioning close to maximum capacity.^42^ This suggests that the functional requirements of a healthy cell to fold CCT substrates are finely tuned in relation to CCT capacity. This is of interest in the context of CCT not being a heat shock protein and the hypothesis that once actin is folded by CCT, it cannot be refolded if it becomes unfolded due to stress. In the case of tubulin, synthesis is tightly regulated and coupled to the microtubule assembly state,^43^, ^44^, ^45^ this mechanism may well contribute to preventing an overloading of the CCT folding capacity. In the case of actin, CCT5 can bind to the co-transcriptional activator of the serum response pathway that co-ordinates actin synthesis with levels of polymerized actin and thus may act as a “safety mechanism” to prevent increased actin synthesis when there is less CCT oligomer assembled.^46^

Mutations in seven of the eight CCT subunits have been reported to be associated with brain malformations of 22 patients, and patient data together with functional studies performed in yeast, worms, and zebrafish indicate a broad range of phenotypes depending on the CCT subunit/mutation.^47^ Patient-derived fibroblast analysis suggested that frameshift mutations affected levels of both the affected subunits and the other subunits, whilst a point mutation most probably affecting the ATPase activity of CCT (CCT5 K176R) did not lead changes in levels of protein and probably has a direct effect on the CCT mechanism.^47^ Expression analysis using patient-derived fibroblasts revealed that proteins involved in for example, cell adhesion, actin nucleation, proteasome and stress chaperones were upregulated, whilst a large proportion of the down-regulated proteins were associated with metabolism.^47^ The authors suggest that the diversity in phenotypes observed could well be a consequence of different mutations affecting the CCT oligomer in different ways and further suggest that even though CCT is ubiquitously expressed, neuronal phenotypes may arise as a consequence of particular requirements of the cytoskeleton during early developmental stages.^47^ It is also possible that CCT subunit-specific mutations may affect interactions with different folding substates and thus lead to diverse phenotypes based of the array of folding substrates affected.

In addition to the folding of substrate proteins, the CCT oligomer also binds to the von Hippel Lindau tumor suppressor protein to promote complex formation with Elongin proteins.^48^ CCT also binds to and affects the phosphorylation levels of the transcription factor STAT3^49^ and interacts with the calcium-activated form of gelsolin.^50^ These observations suggest roles beyond folding for the assembled CCT oligomer. The tomography studies of CCT oligomer distribution in human cells identified both lone CCT oligomers and clusters of the oligomer.^42^ The clusters were not found to accumulate near to the ribosome exit tunnel, but instead approximately 1/3 of clusters were in close proximity to actin filaments.^42^ Whilst this observation may simply be a consequence of actin filaments being highly abundant, it has been shown that the CCT oligomer can affect the rate of actin filament polymerization in in vitro experiments.^51^ Thus, a subset of CCT oligomers may not be actively involved in the folding of newly synthesized polypeptides but instead may act as sequestering complexes or have other roles.

## Monomeric functions of CCT subunits

Long-standing observations, such as non-uniform localization patterns of the CCT subunits in membrane-enclosed organelles being revealed via protein correlation profiling^52^ and some CCT subunits acting as microtubule-associated proteins,^53^ are consistent with additional “moonlighting” functions of CCT subunits when in either subassemblies or as monomers. Uneven expression levels of subunits in yeast would be consistent with some CCT subunits having the potential to be sufficiently abundant to have functions additional to that of being a component of the CCT oligomer^54^ and CCT subunit expression profiles can change in a non-stoichiometric way in tumors (eg, TCGA database). Together, this suggests that under certain circumstances it is possible that there may be altered levels of CCT monomer activity.

There are numerous examples of CCT subunits, when monomeric, displaying functions distinct from protein folding. For example, CCT4 binds to the dynactin component p150^Glued^ and this interaction may influence cell migration.^55^ Furthermore, CCT4 is required for the correct localization of p150^Glued^ to spindle poles during mitosis.^56^ The CCT4:p150^Glued^ interaction was discovered via a yeast two-hybrid screen to identify the CCT4 monomer binding partner involved in the generation of retraction fibres that are formed in cultured mammalian cells transfected with GFP-CCT4^55^ (placement of GFP at the N-termini of CCT subunits hinders oligomer assembly^57^). Similar yeast two-hybrid screens using the other seven CCT subunits as bait identified a CCT monomer interactome consisting of 23 novel interaction partners in total for all eight CCT subunits.^58^ These proteins do not contain common structural features, but more than half of these proteins are involved in processes associated with the cytoskeleton and /or cell cycle.^58^ This study also showed that individual siRNA depletions in cultured mammalian cells lead to distinct cell cycle progression phenotypes, consistent with CCT subunit-specific functions being important at different stages of the cell cycle.^58^

The CCT2 subunit, when monomeric, has been shown to bind solid aggregates and then bind to ATG8 via an interaction sequence that is exposed in monomeric CCT2, allowing for internalization of aggregates in autophagosomes.^59^ This suggests a complex role in proteostasis between folding (CCT oligomer) and targeted degradation (via a monomeric subunit). If the presence of aggregated material can influence CCT oligomer disassembly to provide more free CCT2 subunits, then this would presumably reduce the folding capacity of the cell. Based on the observations of,^60^ that depletion of CCT subunits affects the aggregation of model huntingtin proteins via loss of the autophagy pathway (most probably due to loss of newly folding actin), it is predicted that any shift in the balance of the assembled/monomeric CCT subunits would have complex consequences for protein quality control and proteostasis.

The T400P/R516H heterozygous double mutation in CCT2 causes Leber congenital amaurosis, a disease affecting the retina.^61^ Biochemical studies of the equivalent mutations in yeast CCT2 reveal changes in the ADP-off rate of the CCT oligomer, which may in turn result in a more stable CCT oligomer.^62^ Thus, in addition to potentially altered folding rates, the release of the CCT2 subunit as an autophagy receptor could be affected, leading to increased protein aggregation levels.^59^, ^62^ Taken together, these studies exemplify the complex interplay between oligomer and monomer functions.

## Regulation of the CCT assembly state

**Fig. 2:**
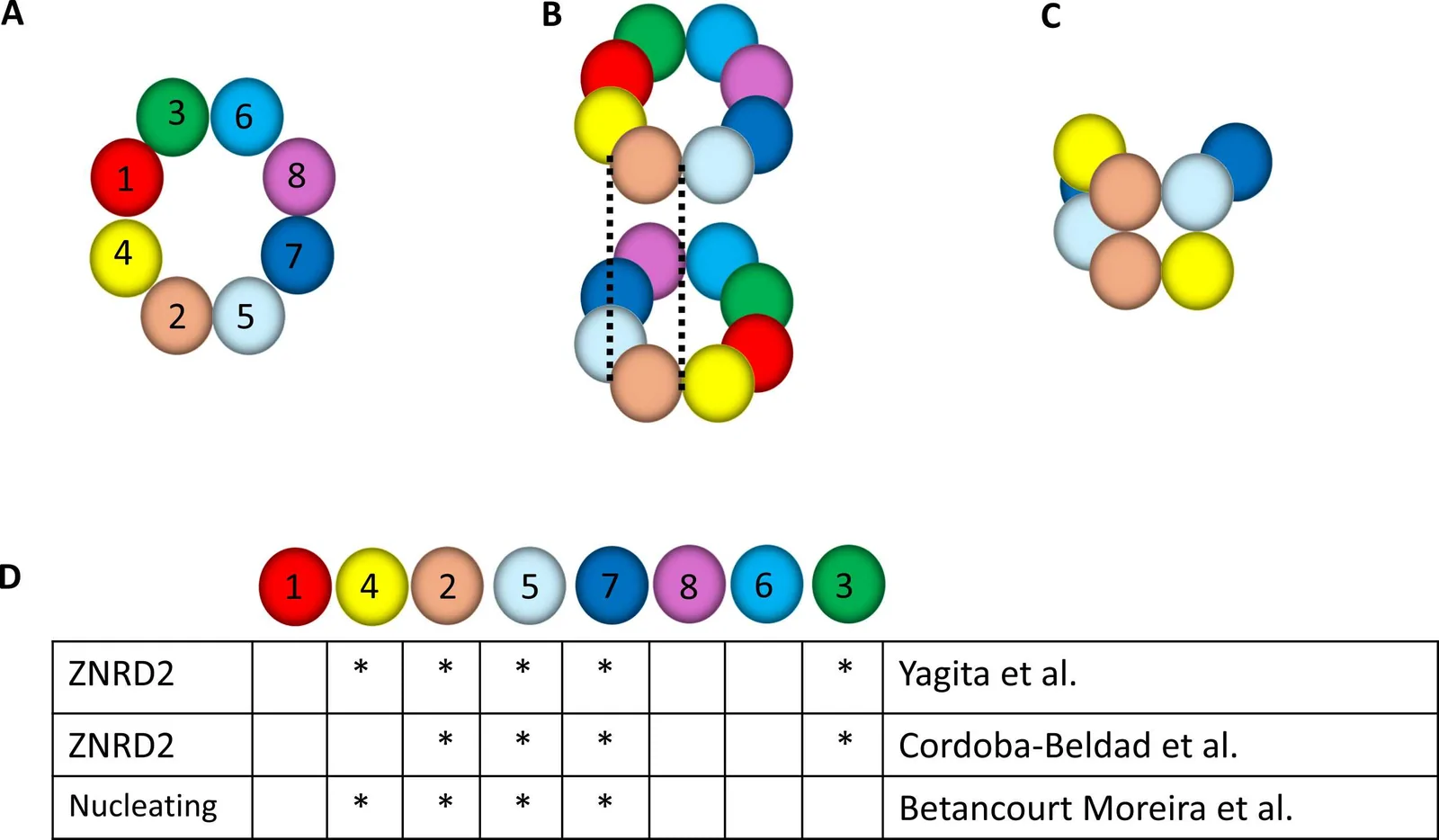
*Components of an assembly intermediate are targeted for degradation as orphan subunits.* (A) The order of subunits within a single chaperonin ring. (B) Orientation of CCT subunits within two rings where both CCT2 subunits are in register with each other. (C) The proposed nucleating assembly intermediate proposed by.^69^ (D) comparison of nucleating assembly and ZNRD2 binding properties.^58^, ^69^, ^70^

In addition to the canonical oligomeric CCT oligomer and free subunits, CCT subunits can form subassemblies, some of which have been reported to exist with interactions between subunits that do not reflect the order of the assembled chaperonin rings,^63^ suggesting sampling of assembly may take place. Additionally, it has been observed that expression of CCT5 in bacteria can produce assemblies.^64^ Although several models of the CCT subunit order have been proposed,^65^, ^66^ the consensus is now that the order proposed by two independent studies using a cross-linking mass spectrometry approach is the true native structure of the CCT oligomer,^17^, ^18^ (illustrated in Figure 2A and B). Furthermore, several electron microscopy studies using antibodies to label CCT subunits have been consistent with only one copy of each subunit being present in each chaperonin ring.^67^, ^68^ This raises the question of how CCT subunits assemble to form oligomers, where each of the eight chaperonin subunits occupy a fixed position within each ring.

A model has been proposed where a hierarchy in CCT subunit assembly occurs, involving the assembly of a core of subunits that spans both chaperonin rings consisting of CCT2, 4, 5 and 7,^69^ (illustrated in Figure 2C). Once a stable core is formed that spans both rings via the CCT2:CCT2 contact, the addition of further subunits can then proceed. The observations leading to this proposal were that in HeLa cells, depletion of each CCT subunit individually led to varied effects on the stability of other subunits depending upon placement within the chaperonin ring, so depletion of CCT2 affects levels of CCT4 also and vice versa.^69^ Similar effects on CCT subunit levels following individual subunit depletion can also be seen in MCF7 cells, suggesting this is a broad mechanism.^49^ Over-expression studies of CCT subunits in insect cells suggested that the balance of subunits can be perturbed, leading to non-canonical subassemblies or even 1 MDa assemblies forming.^69^ However, although it is apparent that non-canonical assemblies can occur,^64^, ^69^ it is considered that such assemblies would be unstable and transient in vivo. The work of^69^ suggests that instead of thermodynamics driving the assembly and thus determining the subunit order of the CCT oligomer, regulation of assembly ensures correctly assembled functional oligomers. Inhibitors of both the proteasome and lysosome/autophagy pathways were found to increase the levels of CCT subunit subcomplexes, suggesting that these pathways will ensure the degradation of incorrect assemblies.^69^

The dynamics of assembly and disassembly of the CCT oligomer and whether other cellular components influence this remain elusive. Purified CCT oligomer from mammalian sources appears to be rather stable and has been the source of CCT for numerous structural studies [eg, ^71^^,^^72^], whilst in the context of the cellular environment it may be more dynamic.^73^ However, yeast CCT oligomer appears to be rather more unstable, requiring additional stabilization during purification protocols.^74^ It has been observed that in lysates prepared from cultured mammalian cells, there is often a proportion of CCT subunits present as monomers/smaller assemblies, as determined by their migration through sucrose density gradients [eg, ^57^]. Some of these small assemblies/monomers will be on assembly/disassembly pathways, whilst some CCT subunits can possess distinct functions when monomers. A recent study identified a pathway for the degradation of monomeric and small assemblies of CCT subunits.^70^ Such a degradation pathway targets “orphan subunits” that are not incorporated into larger assemblies. The study identified the protein ZNRD2 as an adaptor for linking certain CCT subunits to the E3 ligase HERC2 and thus targeting selected CCT subunits for degradation.^70^ Specifically, CCT2, CCT3, CCT4, CCT5, and CCT7 were shown by immunoprecipitation to bind to ZNRD2 and CCT3, CCT4, CCT5, and CCT7 were shown to bind HERC2.^70^ That this pathway appears to only involve a sub-set of CCT subunits could be consistent with a specific need to regulate levels of some but not all CCT subunits. Consistent with such selective binding is the observation that in a yeast two-hybrid screen designed to identify monomeric CCT subunit interaction partners, CCT2, CCT3, CCT5, and CCT7 were also shown to bind ZNRD2.^58^ Four of the CCT subunits shown to bind to ZNRD2,^70^ CCT2, CCT4, CCT5, and CCT7, are neighbors within a single ring and moreover, are the subunits identified as forming the nucleating complex important for CCT assembly^69^ (illustrated in Figure 2D). It is thus possible that ZNRD2-targeted degradation has a role in the removal of assembly complexes that have stalled in their recruitment of other CCT subunits. Alternatively, degradation of these subunits specifically may represent a way to limit oligomer assembly.

The E3 ligase CRL4^DCAF12^ is a ligase involved in assembly quality control and has been shown to bind to two glutamate residues at the C-terminus of CCT5, which are masked in assembled CCT oligomers, and thus CRL4^DCAF12^ is only able to act upon monomeric CCT5.^75^ None of the other seven CCT subunits have two glutamate residues in this region, resulting in this being a subunit-specific interaction. This interaction may represent another way in which the cell can regulate CCT oligomer assembly. How the assembly state of CCT is regulated and how CCT subunit turnover impacts upon this is of great importance for understanding CCT activity, especially as ATP-dependent chaperones are often less expressed during aging/neurodegeneration,^76^ whilst CCT subunits often display enhanced expression in tumors (eg, TGCA database). This latter observation is of relevance in the context of CCT monomer functions (distinct from the folding functions of the CCT oligomer), which may become overrepresented upon increased expression of a particular CCT subunit.

## CCT subunits and cancer

There are numerous reports of altered CCT subunit levels connected to cancer development. For example, using information from publicly available databases, it is observed that in breast cancer samples, six of the CCT subunits increased at the transcriptional level in comparison to normal breast tissue, whilst for seven CCT subunits, overexpression may be associated with poor outcomes in breast cancer.^77^ Similar observations for CCT2 are reported by.^78^ Analysis of multi-omic data reveals that between subtypes of medulloblastoma, CCT subunit levels can be altered at both the protein and mRNA levels, but differences were not observed at the level of DNA methylation.^79^ Furthermore, CCT is suggested as a potential diagnostic tool for rare circulating tumor cells^80^ and for neuroblastoma.^81^ Observations of increased expression of CCT subunits are complex to interpret. For example, CCT5 is highlighted as a protein of prognostic interest in human cancers,^82^ in nasopharyngeal carcinoma^83^ and bladder cancer,^84^ whilst CCT6A was suggested to activate Wnt and Notch pathways in oral squamous cell carcinoma.^85^

**Fig. 3:**
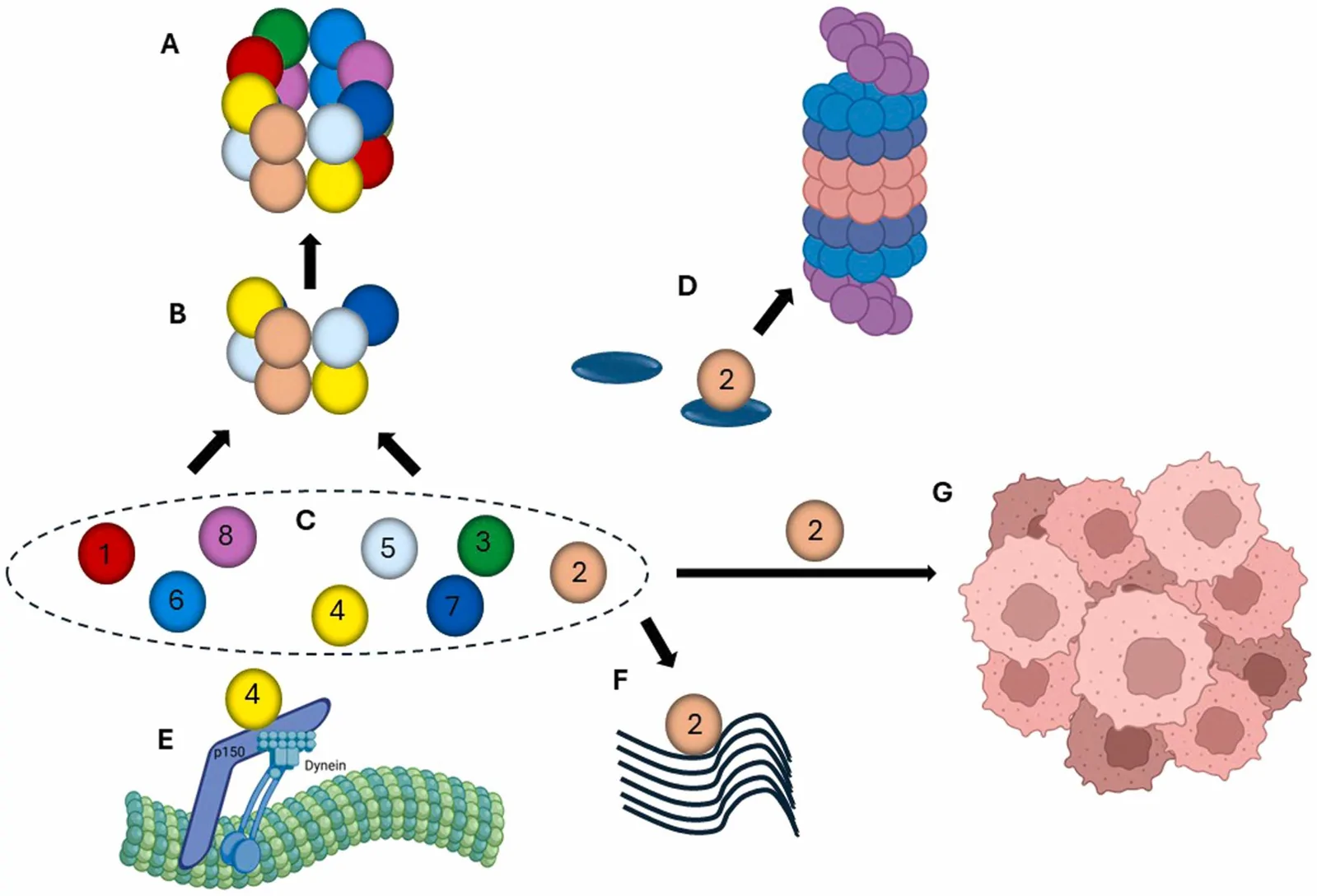
*CCT: complex functions that extend beyond folding.* (A) The CCT oligomer. (B) A proposed cluster of CCT subunits form to create a nucleus for the assembly of the chaperonin rings. (C) The pool of unassembled monomers in the cell, some of which can be targeted for degradation via ZNRD2 binding (D). (E) some CCT subunits display functions when monomeric, eg, CCT4 binding to p^150^Glued. (F) CCT2 may interact with protein aggregates along the pathway to autophagy. (G) Some CCT subunits may be overexpressed in tumors and, for example, CCT2 may be associated with cancer progression. Prepared in part with Biorender.com.

It is not clear if all or just some CCT subunits are critical for promoting carcinogenesis. As CCT forms a multi-subunit assembly where each component is the product of an essential gene, it is probable that assessing the impact of one CCT subunit on for example cell division could reveal both CCT monomer and oligomer dependencies. For example, there are several reports implicating CCT2^86^, ^87^, ^88^ in survival and growth, but these observations could either be specific for monomeric CCT2 or exclusively connected to the folding activities of intact CCT oligomers. Showalter et al.^88^ present the effects of both increasing and decreasing levels of levels of CCT2. They demonstrate that expressing CCT2-Flag in either the non-cancerous breast cell line MCF10A or two breast cancer cell lines (MCF7 and T47D) leads to increases in levels of some other CCT subunits, whilst the endogenous CCT2 appeared reduced.^88^ It is possible that increased levels of CCT2 may promote the formation of the assembly nucleation complex described by.^69^ This could both promote assembly of the CCT oligomer and lead to fewer free CCT subunits available bind to ZNRD2 and thus reduced degradation of some CCT subunits. Interestingly, both^81^, ^88^ report that when cells express CCT2-Flag a reduction in levels of endogenous CCT2 is observed. This could be a consequence of excess CCT2 monomers being potentially degraded by the ZNRD2-mediated pathway and suggests that a complex interplay between assembly and subunit stability/degradation is important for determining CCT subunits levels in the cell. A summary of CCT assembly states and potential functions beyond folding is illustrated in Figure 3.

Reduction of CCT2, lead to reduced tumor growth in a mouse model system and a loss of viability in the breast cancer cell line MDA-MB-231.^88^ This could be explained by the reduction of CCT2 leading to the collapse of the CCT oligomer and was accompanied by reductions in other CCT subunits,^88^ consistent with other observations that targeting one CCT subunit can affect the levels of other subunits and leads to less assembled CCT oligomer.^69^, ^89^ It is possible that as CCT2 forms the core of the assembly complex of the CCT oligomer,^69^ depletion of this subunit could rapidly affect the assembly of oligomers. As the obligate folding substrates actin and tubulin are very abundant and will therefore occupy a substantial proportion of CCT oligomers, it is not surprising that disruption of CCT oligomer folding capacity will have an impact upon processes mediated by actin filaments and microtubules, leading to growth arrest and reduction in cell migration. Additionally, it is possible that levels of the CCT2 subunit directly affect tumor progression, as loss of functional CCT oligomer may mask the significance of loss of specific CCT subunit monomer functions such as the CCT/autophagy functions reported by.^59^

The CCT oligomer can also act as a sequestering complex for the oncogenic transcription factor STAT3^49^ and CCT folds mLST8 and Raptor^71^ and thus influences assembled mTORC. These activities have the potential for CCT oligomer levels to affect numerous cellular processes, many of which are involved in cancer development.

## CCT as a therapeutic target

The interplay between the CCT oligomer for the folding of actin and tubulin and cytoskeletal functions in cell invasion and cell division make CCT worthy of consideration as a therapeutic agent in targeting cancer cells. Furthermore, our increasing knowledge regarding the expression levels and range of functions of the CCT subunits when monomeric suggests that these proteins may well contribute to the overall potential of cells to become cancerous.

In addition to CCT being of interest in cancer cell biology, CCT as a target in parasite and viral disease situations should be considered. CCT from the malaria parasite *Plasmodium falciparum* was shown to vary in abundance at both the transcript and protein level during different stages of the plasmodium life cycle, being most abundant at the feeding and growth (trophozoite) stage.^36^ Analysis of the *P falciparum* CCT interactome revealed a profile of interaction partners including actin and tubulin and WD repeat-containing proteins, indicating that in plasmodium, CCT will contribute to proteostasis by folding the major cytoskeletal components actin and tubulin.^36^ The antihistamine compound clemastine has been shown to bind to CCT4 in *P falciparum* and may affect the stability of the CCT oligomer, resulting in reduced tubulin levels and mitotic spindle defects.^90^ Furthermore, Lu et al^90^ demonstrated that clemastine did not affect the human liver cell line Huh7, indicating the potential for a species-specific targeting of CCT.

The Zika virus protein NS1 has been shown to interact with human CCT in a 293T cell model system, and depletion of CCT2 was shown to reduce Zika virus replication in both HeLa cells and mosquitoes.^91^ These observations of utilization of CCT by a component of the Zika virus suggest that CCT could be a therapeutic target. Furthermore, the reovirus σ3 capsid protein has been shown to bind to CCT and attain a native-like structure upon chaperonin lid closure.^92^ CCT was also shown to be required for the formation of σ3 with its binding partner µ1, implicating CCT in both σ3 folding and assembly.^92^

CCT may play a role in the suppression of aggregates that are associated with neurodegenerative diseases. For example, altered aggregation of polyglutamine tracts, α-synuclein, and tau have been reported. In the case of polyglutamine tracts, a single CCT subunit, CCT1 appears to be sufficient to have an impact,^93^ whist the intact CCT oligomer interacts with α-synuclein.^94^ Recently, it has been reported that the apical domains of CCT3 and CCT7 are sufficient to affect tau aggregation and appear to do so via potentially differing mechanisms based on the variation in morphology of the smaller tau structures observed by electron microscopy in the presence of the CCT3 and CCT7 apical domains.^95^ Thus, altered CCT levels or even application of an apical domain may present future therapeutic approaches for combating aggregation-driven pathologies.

## Conclusion

The CCT oligomer plays a central role in protein quality control as an essential molecular chaperone for the highly abundant cytoskeleton proteins actin and tubulin. This, together with the folding of some less abundant, possibly non-obligate, substrates and sequestering activities, leads to the CCT oligomer being estimated to be functioning close to capacity in a growing cell. Recent advances in the field identify CCT subunit monomer activities and address how the CCT oligomer assembles and reveal that selected CCT subunits can be targeted for degradation.

Thus, we are presented with a complex protein quality control machine balancing a range of functions with the assembly state of CCT. Perturbations in this balance, such as altered CCT subunit expression levels or changes to the assembly state, have the potential to have major implications on the contribution of CCT to proteostasis.

## CRediT authorship contribution statement

**Julie Grantham:** Writing – review & editing, Writing – original draft.

## Declaration of Competing Interest

The authors declare that they have no known competing financial interests or personal relationships that could have appeared to influence the work reported in this paper.

## Data Availability

No data was used for the research described in the article.
